# Spontaneous Forniceal Rupture in Pregnancy

**DOI:** 10.1155/2015/379061

**Published:** 2015-01-11

**Authors:** Roshni Upputalla, Robert M. Moore, Belinda Jim

**Affiliations:** ^1^Department of Nephrology/Medicine, Jacobi Medical Center, Albert Einstein College of Medicine, Bronx, NY 10461, USA; ^2^Department of Obstetrics and Gynecology, Jacobi Medical Center, Albert Einstein College of Medicine, Bronx, NY 10461, USA

## Abstract

Forniceal rupture is a rare event in pregnancy. We report a case of a 26-year-old primigravid woman who experienced a forniceal rupture at 23 weeks of gestation with no inciting cause except for pregnancy. Pregnancy is associated with ureteral compression due to increase in pelvic vasculature with the right ureter more dilated due to anatomic reasons. Hormones such as prostaglandins and progesterone render the ureter more distensible to allow for pressure build-up and an obstructive picture at the collecting system. We will discuss physiologic changes in pregnancies that predispose to this uncommon phenomenon and the most up-to-date management strategies.

## 1. Introduction

Forniceal rupture of the kidney in pregnancy is an uncommon entity. Pregnancy induced physiological changes predispose to this condition. Due to its relative rarity, management and treatment of this condition is often unclear.

## 2. Case Presentation

**Figure 1:**
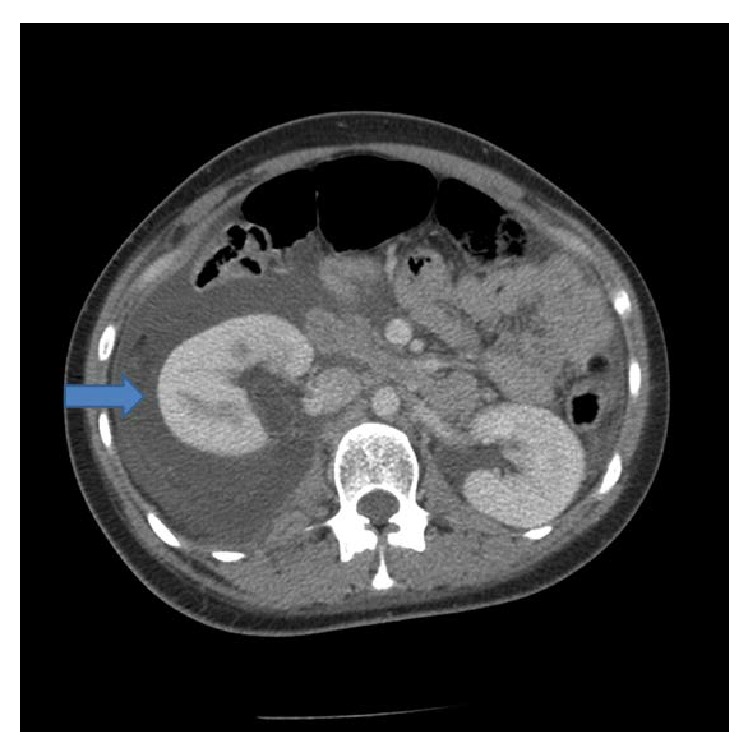
CT scan of abdomen on admission. Blue arrow indicates presence of urinoma.

We present a case of a 26-year-old pregnant female (G_1_P_0_) who presents at 23 weeks of gestation complaining of acute right sided flank pain for one day with no other associated symptoms such as fever or dysuria. Her physical exam was remarkable for right costovertebral angle tenderness. The patient's chemistry and hematologic laboratory values remained normal (Table 1). She was admitted with an impression of pyelonephritis and was started on antibiotics. Imaging studies including renal ultrasound, CT (Figure 1), and MRI were performed which revealed a right forniceal rupture with no evidence of nephrolithiasis. The initial aspiration of the fluid returned a sterile culture. She improved symptomatically with conservative therapy and was discharged home. However, four days later, she returned to a different hospital with similar complaints of right flank pain. A repeat CT scan revealed a urinoma measuring 17.5 cm. The urinoma was subsequently drained followed by the placement of a nephrostomy tube. The patient improved symptomatically and was discharged home. She was to follow up in outpatient urology, renal, and obstetric clinics. The patient continued to be symptom-free for the rest of the pregnancy and delivered at 37 weeks via a spontaneous vaginal delivery. The nephrostomy tube remained in through the remainder of the pregnancy with careful monitoring for infection; it was removed successfully two weeks postpartum.

## 3. Discussion

To qualify as a spontaneous forniceal rupture, the following criteria must be met: the absences of recent ureteric instrumentation, surgery, external trauma, a destructive kidney lesion, kidney stones, or external compression [1]. In a retrospective review of 108 cases of forniceal rupture diagnosed by CT scan, the causes were ureteric stones in 80 cases (74.1%), malignant extrinsic ureteric compression in nine cases (8.3%), benign extrinsic ureteric compression in two cases (1.9%), pelvic-ureteric junction obstruction in two cases (1.9%), vesicoureteric junction obstruction in one case (0.9%), bladder outlet obstruction in one case (0.9%), and iatrogenic causes in four cases (3.7%) [2]. In fact, no definitive cause was found in nine cases (8.3%). Pregnancy has been described as a much more rare cause of forniceal rupture [3]. Pregnancy with a solitary kidney, however, has also been reported to result in forniceal rupture [4].

It is surmised that forniceal rupture is a safety valve for alleviation of increased intrapelvic pressure. According to Laplace's law (Tension = Pressure × Radius) the tensile stress that accumulates within a dilated collecting system would increase with size, thereby causing earlier forniceal rupture in a more dilated system [2]. A pressure that exceeds the tensile strength of forniceal tissues leads to rupture and extravasation of urine. Ultimately, this phenomenon is meant to be renoprotective by decreasing pressure in the collecting system [5].

The state of pregnancy results in physiologic hormonal and hemodynamic changes of kidney size, structure, and function. Both kidneys increase in size by 1–1.5 cm due to the increase in the renal vascular volume, hence increasing the glomerular filtrate rate by 50% [6]. The ureters are retroperitoneal structures that go from the renal pelvis to the bladder. They are around 25 to 30 cm in length from the renal pelvis to the trigone of the bladder. They are divided by the pelvic brim into abdominal and pelvic segments, each of which is around 12 to 15 cm in length. That physiologic hydronephrosis and hydroureters that occur in pregnancy have been well-described for more than 200 years. Both Morgagni in 1761 and Rayer in 1839 discerned from postmortem examination that the uterus compressing the ureters produced retention of urine in the kidneys. This then causes dilatation of structures such as ureters, pelvis, and calyces, which results in a delay in the excretion of urine [7]. With increase in the pelvic vasculature in pregnancy, ureteral compression occurs, more on the right because of anatomic relationship of right ureter to less distensible right iliac artery and right ovarian blood vessels. The ureters and renal pelvis dilate more so on the right than left (up to 80%) [7]. The dilatation involves only the renal pelvis and the abdominal ureter and is evident by the third trimester of pregnancy in almost 90% of pregnant patients. This effect usually resolves in half of the females within two days of delivery.

Apart from mechanical reasons, endocrine factors also contribute to ureteropelvic distension. Hormones such as progesterone and prostaglandins can cause diminished tone and peristalsis of the ureter. This allows for small increments of extraluminal pressure to produce substantial reductions in urine flow and distension of the collecting system proximal to a point of obstruction. Interestingly, high-dose hormonal therapy failed to produce ureteropelvic dilatation reliably, suggesting that hormonal causes alone are not enough to explain the structural alterations [8].

With all the above features, there is progressive dilatation of the renal pelvis and the risk of forniceal rupture. The incidence for rupture will be highest at points where there is scarring and infection due to decreased structural integrity. In the absence of these factors, the site of rupture is unclear but may be traced to the calyx or pelvis where a collection of urine may be found.

The clinical presentation of a forniceal rupture in pregnancy may be confused with many abdominal processes, including but not limited to cholecystitis, hepatitis, appendicitis, pyelonephritis, uterine rupture, abruption placentae, and more. Laboratory testing is usually not very helpful in delineating the exact etiology, though normal liver function tests may help to rule out liver pathologies. Imaging of the collecting system, initially with an ultrasound, usually followed with a CT or MRI scan, would be more definitive. A limited excretory urogram is not commonly performed during pregnancy but may help to delineate the site and nature of the rupture or obstruction, the amount of extravasation, and the function of the kidneys.

Management is individualized and depends on the location and type of extravasation. Hwang et al. report the use of serial ultrasonography to detect, monitor, and manage the rupture [9]. On ultrasound, the presence of perinephric fluid may be difficult to distinguish between a urinoma and a hematoma, with helpful hints from the presence of internal echoes or septations indicating the latter. However, oftentimes, an MRI is necessarily performed to view the characteristic high-intensity signals of acute hematoma on T1-images [10]. If the rupture occurred through the renal parenchyma, then surgical exploration is necessary because of the associated hemorrhage. A partial or total nephrectomy may be necessary to control the bleeding [11]. In cases where the collecting system is ruptured, the goal is to alleviate the outflow obstruction. For the patient with a gravid uterus that compresses the ureter, placement of a double J ureteral stent [4] or a nephrostomy tube should relieve the pressure [10]. Appropriate antibiotic coverage and close follow-up are required until definitive treatment is possible, that is, the delivery of the child. It is recommended to change ureteral stents and nephrostomy tubes every 4–6 weeks during pregnancy [12]. Ureteral stents, in particular, have a high risk of encrustation. Why this phenomenon is increased during pregnancy is not completely clear but may be related to the hypercalciuric and hyperuricosuric states that are associated with pregnancy [13]. Frequent urinary tract infections or asymptomatic bacteriuria may exacerbate this as well. Prolonged nephrostomies are associated with increased risk of infection. Since radiation exposure is a concern, it is possible to perform ultrasound-guided ureteral stent placement with local anesthesia and intravenous sedation [14]. However, if this expertise is not available, placement under pulsed fluoroscopy will help to minimize radiation exposure. Oesterling et al. reported one case report of a spontaneous forniceal rupture during pregnancy that was managed only with the temporary insertion of a ureteral catheter for 72 hours [11]. A retrograde pyelogram conducted after removal of the catheter showed no further extravasation and the patient remained asymptomatic for the remainder of her pregnancy. The authors recommend that a short trial of ureteral catheter placement of 48 to 72 hours may be tried; if recurrence is noted, a self-retaining indwelling catheter should be inserted for the duration of the pregnancy. Hwang et al. performed a retrograde ureteral catheterization and were able to relieve the patient's flank pain and rapidly resorb the perinephric urinoma, which suggested that there was an open communication between the site of rupture and the urinoma [9]. Conservative management is usually adequate; however, should the patient demonstrate clinical deterioration in the form of decreasing hemoglobin or increasing in the size of collection, nephrectomy may be necessary to control the hemorrhage provided the other kidney is normal [4].

In conclusion, though ureteral dilatation is common in pregnancy, forniceal rupture is not. Management of this condition ranges from temporary insertion of a ureteral catheter to total nephrectomy depending on the site and severity of rupture. There have not been reports on whether the rupture recurs in subsequent pregnancies, though, given the anatomic damage, the risk should be higher. Careful postpartum follow-up with nephrology and urology would be crucial.

## Figures and Tables

**Table 1:** Admission laboratory values.

Laboratory values	Admission
Sodium (mmol/L)	142
Potassium (mmol/L)	4.2
Chloride (mmol/L)	108
Bicarbonate (mmol/L)	24.4
BUN (mmol/L)	3.21
Serum creatinine (*μ*mol/L)	44.2
WBC (/nL)	10.1
Hemoglobin (g/dL)	11.6
Hematocrit (%)	33.7
Platelets (/nL)	164
